# Insights into geriatric health: primary sarcopenia and innate immunity dynamics, examining SARC-F, serum TLR 4, TLR 9, and resolvin levels

**DOI:** 10.1007/s11739-024-03678-5

**Published:** 2024-06-23

**Authors:** Seyda Bilgin, Veysel Suzan, Suna Avci, Hakan Yavuzer, Ibrahim Murat Bolayirli, Alper Doventas, Deniz Suna Erdincler

**Affiliations:** 1 Division of Geriatric Medicine, Department of Internal Medicine, Cerrahpasa Medical Faculty, Istanbul University-Cerrahpasa, Cerrahpaşa Mahallesi Kocamustafapaşa Caddesi No:34/E Fatih, Istanbul, Turkey; 2 Department of Biochemistry, Cerrahpasa Medical Faculty, Istanbul University-Cerrahpasa, Istanbul, Turkey; 3 Department of Immunology, Institute of Health Sciences, Istanbul University, Istanbul, Turkey

**Keywords:** Sarcopenia, Aging, SARC, F, TLR9, Innate Immunity

## Abstract

The aim of this study is to evaluate the relationship between serum TLR (Toll Like Receptor) 4, 9 and Resolvin E1 levels and primary sarcopenia in geriatric patients and to compare the diagnostic accuracy of these biomarkers with the SARC-F score. A total of 88 patients aged 65 years and older were evaluated in the study. Comorbidities and geriatric syndromes were identified and patients with secondary sarcopenia were excluded. EWGSOP2 criteria were used as diagnostic criteria for sarcopenia and SARC-F questionnaire was used to find individuals at risk for sarcopenia. Serum TLR 4, 9 and Resolvin E1 levels were analyzed by ELISA. There were no significant differences between the two groups in terms of age and gender (p = 0.654 and p = 1.000, respectively). SARC-F, serum TLR 9 and Resolvin E1 were significantly higher in the sarcopenia group compared to the non-sarcopenia group (p < 0.001, p < 0.001 and p = 0.040, respectively). Statistically significant parameters were evaluated by multiple regression analysis. TLR 9 and SARC-F score were both found to be associated with sarcopenia in multivariate logistic regression analysis [Odds ratio (OR) 3145, (95%) confidence interval (CI) 5.9–1,652,888.3, p = 0.012; OR 4.788, (95%) CI 2.148–10.672, p < 0.001, respectively]. ROC curve analysis showed that the area under the ROC curve (AUC) for TLR 9 and SARC-F was 0.896 (p < 0.001) and 0.943 (p < 0.001), respectively. Although this study supports the use of the SARC-F questionnaire in daily practice, serum TLR 9 levels may be an alternative to SARC-F in cases where SARC-F is not feasible.

**Supplementary Information:**

The online version contains supplementary material available at 10.1007/s11739-024-03678-5.

## Introduction

After the fourth decade of life, muscle mass and strength decrease in both men and women [1]. Various operational definitions of sarcopenia have been published over the years. However, the European Working Group on Sarcopenia in the Elderly (EWGSOP) consensus definition published in 2010 and updated in 2019 (i.e., EWGSOP2) appears to be the most widely adopted and used definition worldwide. In clinical practice, EWGSOP2 recommends using the SARC-F (Strength, Assisted Walking, Chair Standing, Stair Climbing and Falling) questionnaire to find people at risk of sarcopenia. After the screening test, signs of low muscle strength indicate possible sarcopenia; sarcopenia is diagnosed by adding low muscle mass to low muscle strength. In addition, sarcopenia is considered severe when low physical performance is present [2]. Sarcopenia is termed "primary" (age-related) when no underlying cause can be found and "secondary" when causal factors other than (or in addition to) aging are evident. The most common causes of secondary sarcopenia are catabolic processes, changes in sex hormones, malnutrition with low protein intake, inactivity and inflammatory diseases [3]. Sarcopenia is considered a sign of frailty that plays a key role in the development of frailty [4, 5].

Toll-Like Receptors 4 and 9 (TLR4,9) are pattern recognition receptors because this receptor family is activated by recognizing biological molecules with common structures or motifs. In mammals, there are at least ten types of TLRs responsible for extracellular and intracellular activities [12]. These receptors can recognize common shared motifs (PAMPs) of exogenous pathogens as well as common motifs of damage markers (DAMPs) resulting from intracellular stress and inflammation [13]. The TLR family are members of the innate immunity and are activated in a variety of conditions ranging from infection to chronic inflammation and tumoral immunity. In addition, in studies on lipid mediators in the inflammatory response, omega-3 PUFA-derived mediators (Resolvin E1, D-series resolvins and E-series resolvins such as protectins) are secreted from inflammatory exudate and show anti-inflammatory effects through resorption of inflammation [16]. Resolvin E1 targets neutrophils, dendritic cells, macrophages, platelets and adhesion molecules. In animal models, Resolvin E1 has been shown to have a potent protective effect against leukocyte-mediated tissue damage and excessive proinflammatory gene expression in the pathogenesis of inflammatory diseases. Recent studies have shown that Resolvin E1 plays a role in the etiopathogenesis of inappropriate inflammatory responses such as rheumatoid arthritis, multiple sclerosis, bronchial asthma, retinopathies, periodontal diseases and inflammatory bowel disease [17–19].

When we looked at the literature, we saw that numerous cytokine and other biomarker studies have been performed in sarcopenia. At this point, the selection of these biomarkers (TLR4, TLR9 and Resolvin E1), especially the extreme scarcity of the studies and the fact that all of them are animal studies, is to examine whether the possible hypothetical mechanisms of a condition such as primary sarcopenia, which is less confounding than secondary sarcopenia, are valid in humans. The aim of this study was to evaluate the relationship between markers involved in different aspects of inflammation such as serum TLR4, TLR9 and Resolvin E1 levels and primary sarcopenia in geriatric patients and to compare the diagnostic accuracy of these biomarkers with the SARC-F score.

## Methods

### Study design, participants, and ethical considerations:

The study population consisted of 44 individuals with sarcopenia and 44 individuals without sarcopenia aged 65 years and older receiving routine medical care in the geriatrics outpatient clinic of a university hospital. All participants were documented about their comorbid diseases and geriatric syndromes. Bed-bound patients and patients with rheumatoid arthritis, advanced organ failure, HIV infection, malignancy, inflammatory bowel disease, chronic kidney disease, chronic liver disease, advanced COPD, morbid obesity, malabsorption, steroid use, thyroid dysfunction, malnutrition, anemia, acute and chronic infection were excluded. Laboratory data of the patients included in the study were obtained from the electronic database of our hospital. The study protocol was approved by the Local Research Ethics Committee. This study was supported by Istanbul University-Cerrahpaşa Research Fund (Project Number: 37184).

### Assessment of frailty and comorbidity index

Clinical frailty index (CCI) (Clinical Frailty Scale / Rockwood Frailty Index) was used to evaluate the frailty status of both groups [6, 7]. This index, derived from the Canadian Study of Health and Aging, is a scale that scores patients' daily activity status, whether they experience symptoms of disease and whether they need help from others on a scale of 1–7, and people with higher scores are considered to be at higher risk for disease-related complications. In the CCI scoring system, patients with a score of 1–3 are generally considered to be in good condition, those with a score of 4 are considered to be susceptible to frailty, and those with a score of 5–7 are considered to be frail [6, 7]. In our study, CCI assessment was performed by the physician in consultation with the patient. In addition, comorbidities were also evaluated in both groups by applying Charlson's comorbidity scale. The Charlson Comorbidity Index is a method of categorising patients' comorbidities based on International Classification of Diseases (ICD) diagnosis codes found in administrative data such as hospital summary data. Each category of comorbidity has a corresponding burden (between 1 and 6). The sum of all burdens results in a single comorbidity score for a patient. A score of zero indicates no comorbidities. The higher the score, the higher the probability that the predicted outcome will result in mortality or higher resource utilisation [8]. Drug use was questioned in detail and the number of drugs used was also recorded.

### Assessment of nutrition status

Nutritional status was assessed BMI, MNA-LF and MNA-SF, which allow us to diagnose normal nutritional status (NNS) as well as the risk of undernutrition and altered nutritional status due to calorie and protein deficiencies, such as malnutrition [9, 10]. The final MNA-LF score ranges from 0 to 30 and is categorised as follows: < 17 malnourished, 17–23.5 at risk of malnutrition and 24–30 normal nutritional status. The final MNA-SF score ranges from 0 to 14 and is categorised as follows: 0–7 malnourished, 8–11 at risk of malnutrition and 12–14 normal nutritional status. BMI value was calculated in kg/m^2^. At this point, patients with malnutrition and severe morbid obesity were excluded from the study.

### Sarcopenia definition

EWGSOP2 criteria were used for the diagnosis of sarcopenia and determination of cutoff values [2]. There are five questions in the SARC-F questionnaire used for sarcopenia case-finding. Participants were asked to answer these five questions with one of the options ‘0 = no difficulty, 1 = some, and 2 = a lot or unable to do’. The cut-off value in the SARC-F questionnaire was accepted as ≥ 4. Muscle strength was measured with a hand dynamometer (Takei® TKK 5401 model, Takei Scientific Instruments Co., Tokyo, Japan). Individuals sat on a chair, with elbows on the table and arms parallel in 90-degree flexion; measurements were made 3 times with 1 min rest periods for both right and left hands. The highest value of the handgrip strength is recorded. For a positive handgrip strength test, cut-off value was accepted as 27 kg for men and under 16 kg for women. Skeletal muscle mass index (SMMI) was measured by the bioelectrical impedance analysis (BIA) device (Tanita Body Composition Analyzer® TBF-300 model, Tanita Co., Tokyo, Japan) in kg/m^2^, after 12 h of fasting and its cut-off value was < 7.0 kg/m^2^ in males and < 5.5 kg/m^2^ in females. Physical performance was evaluated by general gait speed. For the 6 m walking test, the patient walked 6 m in standing position and this time was recorded in seconds with a stopwatch. Gait speed cut-off value was accepted as ≤ 0.8 m/s.

### Collection of blood samples

Fasting venous blood samples were drawn in the morning after an overnight fasting (10–12 h) to tubes without anticoagulants. The blood samples were centrifuged for 10 min at 4000 rpm at 4 °C. For the determination of TLR 4, TLR 9, and Resolvin E1, serum aliquots were frozen and stored at – 70 °C immediately until further analysis. Hemolyzed and lipemic samples were excluded. Preanalytical processes were followed in coordination by geriatricians and clinical biochemists.

### Biochemical analysis

TLR 4, TLR 9 and Resolvin E1, levels were determined in duplicate by the enzyme linked immunoabsorbent assay (ELISA) using commercial kits (Elabscience Human TLR 4 and TLR 9 Elisa kit 96 test, BT-LAB Human Resolvin E1 Elisa kit 96 test). For these parameters, inter- and intraassay coefficients of variation (CV) values were < 10% and < 8% respectively.

### Statistical analysis

A sample size of 40 patients per group was calculated to provide 80% power to detect the expected difference between the two groups for serum TLR4, TLR9 and Resolvin E1 levels. Categorical variables are presented as frequencies. Numerical variables with normal distribution are presented as mean ± standard deviation, and those without normal distribution are presented as median and interquartile ranges (IQR). Categorical variables were analysed using the Chi-square test. Continuous variables were analysed using unpaired Student's t test or Mann–Whitney U test. Statistically significant parameters were also evaluated by multivariate logistic regression analysis. Receiver operating characteristic (ROC) curve analysis was performed to assess diagnostic accuracy. ROC curves were plotted to visualise the power of TLR 9 and the SARC-F questionnaire in the diagnostic accuracy of sarcopenia. The area under the curve (AUC) was then given with a 95% confidence interval and a P value < 0.05 was considered statistically significant.

## Results

In our study, there were 44 patients (29 females) with primary sarcopenia and 44 patients (29 females) without primary sarcopenia. The mean age of the sarcopenia group was 74.93 ± 6.31 years and the mean age of the non-sarcopenia group was 74.40 ± 4.42 years. There was no significant difference in gender and age between the two groups (p = 1.000, p = 0.654). Grip strength, muscle mass, gait speed and BMI were significantly lower in the sarcopenia group (p < 0.001, p < 0.001, p < 0.001, p < 0.001). SARC-F, FRAIL questionnaire scores were significantly higher in the sarcopenia group (p < 0.001, p < 0.001), while MNA-SF, MNA-LF questionnaire scores were significantly higher in the non-sarcopenia group (p < 0.00, p < 0.001), but there was no malnutrition in both groups. There was no significant difference between the two groups in terms of number of medications, number of chronic diseases and Charlson Comorbidity Index (p = 0.421, p = 0.219, 0.360). A history of atrial fibrillation was significantly higher in sarcopenia patients (p = 0.029), while there was no significant difference between the two groups in terms of coronary artery disease, diabetes mellitus, hypertension, heart failure, hypothyroidism, chronic obstructive pulmonary disease, benign prostatic hyperplasia, hyperlipidaemia, incontinence and asthma (p = 0.616, p = 0.325, p = 0.131, p = 1.000, p = 0.214, p = 1.000, p = 0.713, p = 0.113, p = 0.494, p = 1.00 respectively). Detailed analysis is shown in Table 1.

**Table 1:** Demographic data, chronic diseases and anthropometric measurements of the group with and without sarcopenia

	Sarcopenia	No Sarcopenia	p
Number of Patients	44	44	
Gender (Female/Male)***	29/15	29/15	1.000
Age*	74.93 ± 6.31	74.40 ± 4.42	0.654
Grip strength*	18.5 ± 5.05	27.25 ± 6.11	** < 0.001**
Muscle mass (SMMI)*	5.17 ± 0.68	6.4 ± 0.76	** < 0.001**
Gait speed (m/sn)*	0.77 ± 0.21	1.13 ± 0.19	** < 0.001**
SARC-F**	3 (3–4)	0 (0–1)	** < 0.001**
FRAİL*	2.54 ± 0.81	1.61 ± 0.65	** < 0.001**
MNA-SF*	12.70 ± 1.09	13.52 ± 0.66	** < 0.001**
MNA-LF*	26.42 ± 1.69	28.21 ± 1.19	** < 0.001**
BMI*	25.18 ± 4.01	30.58 ± 4.94	** < 0.001**
Charlson Comorbidity Index**	1 (0–1)	0 (0–1)	0.360
Number of Medications*	2.06 ± 1.61	2.36 ± 1.80	0.421
Number of Chronic Diseases*	1.52 ± 1.08	1.84 ± 1.31	0.219
Coronary Artery Disease***	3 (%6.8)	1 (%2.2)	0.616
Diyabetes Mellitus***	9 (%20.4)	13 (%29.5)	0.325
Hypertension***	22 (%50)	29 (%65.9)	0.131
Heart Failure***	0 (%0)	0 (%0)	1.000
Hypothyroidism***	4 (%9)	8 (%18)	0.214
Chronic Obstructive Pulmonary Disease***	1 (%2.2)	1 (%2.2)	1.000
Benign Prostatic Hyperplasia***	3 (%6.8)	5 (%11.3)	0.713
Atrial Fibrillation***	16 (%36.3)	7 (%15.9)	**0.029**
Hyperlipidaemia***	6 (%13.6)	12(%27.2)	0.113
Incontinence***	0 (%0)	2 (%4.5)	0.494
Asthma***	2 (%4.5)	2(%4.5)	1.000

*SMMI* skeletal muscle mass index, *MNA-SF* Mini Nutritional Assessment-Short Form, *MNA-LF* Mini Nutritional Assessment-Long Form, *BMI* Body Mass Index

^*^Mean ± standard deviation **median (25th percentile-75th percentile) ***n (%)

Statistically significant p values are indicated as bold

Serum TLR 9 and Resolvin E1 levels were statistically significantly higher in the sarcopenia group compared to the non-sarcopenia group (p < 0.001, p = 0.040), while TLR4 levels were higher in the sarcopenia group, but this increase was not statistically significant (p = 0.742) (Table 2, Supplementary Fig. 1).

**Table 2:** Comparison of serum TLR 4, TLR 9 and Resolvin E1 levels in sarcopenia and non-sarcopenia groups

	Sarcopenia	No Sarcopenia	p
TLR-9 (ng/mL) *	0.95 (0.83–1.35)	0.60 (0.52–0.79)	** < 0.001**
TLR-4 (pg/mL)*	130.45 (89.60 -169.95)	121.35 (87.23–173.97)	0.742
ResolvinE1 (ng/L)*	159.21 (137.1–176.97)	148.60 (125.27–166.80)	**0.040**

*TLR* toll like receptor

Data* are shown as median (25th percentile-75th percentile)

Statistically significant p values are indicated as bold

While serum haemoglobin level was statistically significantly lower in the group with sarcopenia compared to the group without sarcopenia (p = 0.010), there was no statistical significance between the two groups in terms of neutrophil, monocyte, platelet, lymphocyte count, haemoglobin, serum creatine, vit-D, B12, folic acid, CRP, sedimentation rate, fasting glucose, Hba1-c, ferritin and transferrin saturation (p = 0. 084, p = 0.387, p = 0.639, p = 0.423, p = 0.230, p = 0.777, p = 0.560, p = 0.657, p = 0.574, p = 0.616, p = 0.484, p = 0.741, p = 0.595, p = 0.095 respectively) (Supplementary Table 1).

Multivariate logistic regression analysis was performed for atrial fibrillation, TLR 9, SARC-F, Resolvin E1 and haemoglobin levels which were statistically significant between sarcopenia and non-sarcopenia groups. As a result of this analysis, TLR 9 and SARC-F score were found to have a significant effect on sarcopenia [Odds ratio (OR) 3145, (95%) confidence interval (CI) 5.9–1,652,888.3, p = 0.012; OR 4.788, (95%) CI 2.148–10.672, p < 0.001, respectively]. Detailed analysis is shown in Table 3.

**Table 3:** Multivariate logistic regression analysis for the diagnosis of sarcopenia

	Odds Ratio (95% CI)	p
TLR-9	3145 (5.9–1,652,888.3)	**0.012**
Resolvin E1	1.000 (0.996–1.004)	0.964
Haemoglobin	0.801 (0.383 – 1.673)	0.555
AF	0.488 (0.064–3.709)	0.488
SARC-F	4.788 ( 2.148 – 10.672)	** < 0.001**

*TLR* Toll Like Receptor, *CI* confidence interval, *AF* atrial fibrillation

Statistically significant p values are indicated as bold

ROC curve analysis showed that the area under the ROC curve (AUC) of TLR 9, SARC-F and Resolvin levels were 0.896, 0.943 and 0.627, respectively. When the cut-off value of TLR 9 was 0.815 ng/ml, the sensitivity was 79.5% and specificity 84.1%; when the cut-off value of SARC-F was 4 points, the sensitivity was 70.5% and specificity 97.7%; when the cut-off value of Resolvin was 153.0 ng/L, the sensitivity was 61.4% and specificity 59.1%. The comparison of sensitivity, specificity, AUC, cut-off value and ROC curves of TLR 9, SARC-F and Resolvin levels are shown in Table 4 and Supplementary Fig. 2.

**Table 4:** Receiver operating characteristic curve (ROC) analysis and relative values of TLR9, Resolvin E1 and SARC-F for sarcopenia

	Sensitivity (%)	Specifity (%)	AUC	Cutoff	Asymptotic significance
TLR-9	79.5	84.1	0.896	0.815 ng/ml	** < 0.001**
Resolvin E1	61.4	59.1	0.627	153.0 ng/L	**0.040**
SARC-F	70.5	97.7	0.943	4	** < 0.001**

*TLR* toll like receptor, *AUC* area under the curve

Statistically significant p values are indicated as bold

## Discussion

As stated in EWGSOP2, low muscle strength and muscle mass are necessary for the diagnosis of sarcopenia [2]. However, appropriate equipment, technical staff and sufficient time are required to perform these measurements. In 2019, EWGSOP2 recommended the use of the SARC-F questionnaire for screening and identified a new strategy to reach sarcopenic individuals more easily. This opened new doors for the investigation of alternative screening methods such as biomarkers. In this study, we evaluated the relationship between serum Resolvin E1, TLR 4, TLR 9 and SARC-F questionnaire and primary sarcopenia.

**Fig. 1:**
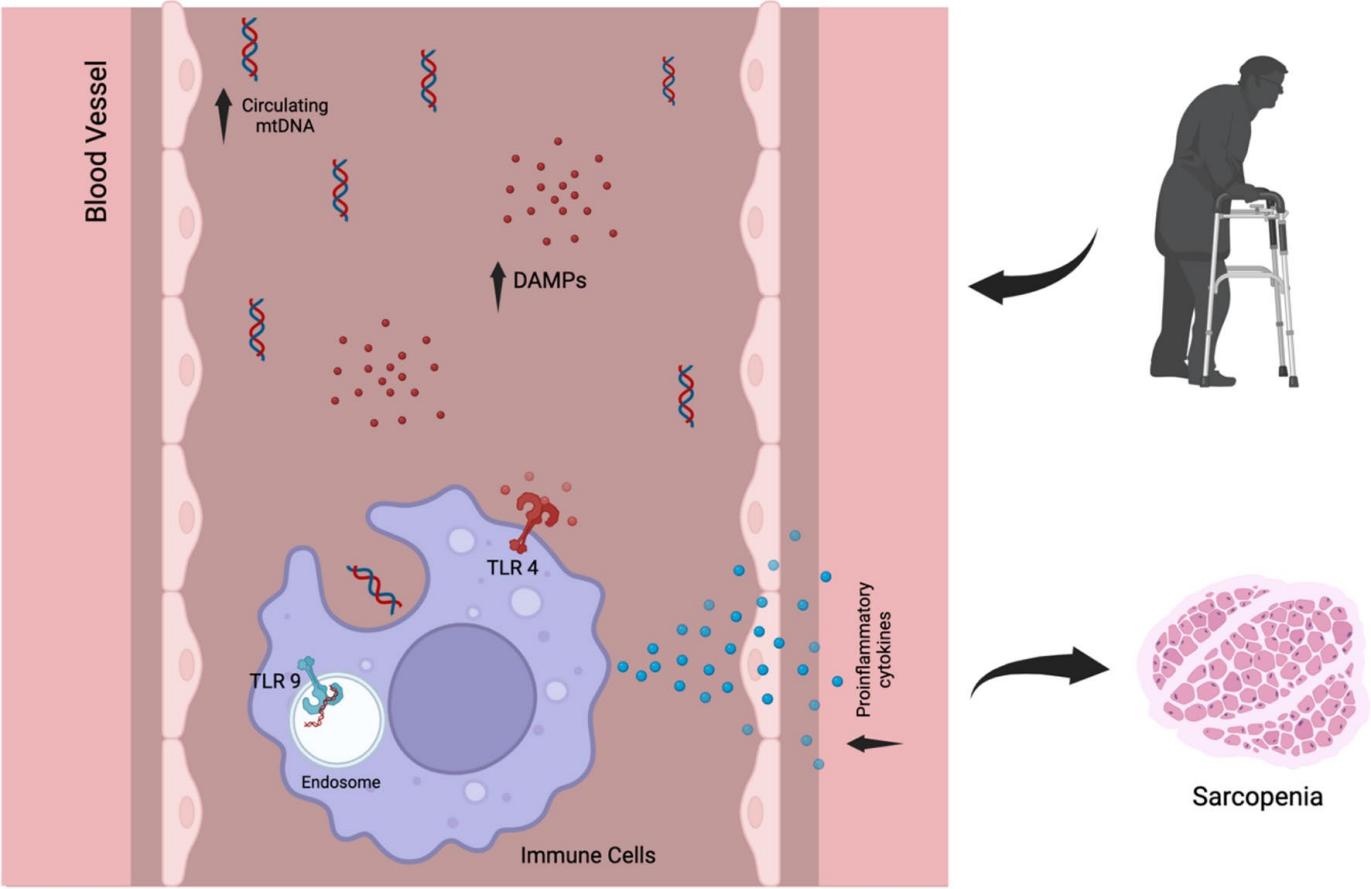
Sarcopenia and TLR 9. *DAMPs* damage associated molecular pattern, *TLR* toll like receptor, *mtDNA* mitochondrial DNA

Inflammatory cytokines are known to accelerate muscle loss, stimulate protein catabolism and suppress muscle synthesis. The relationship between sarcopenia and inflammation is not fully understood. It is predicted that there may be low-grade inflammation in the onset and progression of sarcopenia [11]. Soluble Toll-Like Receptors (TLRs) are released into the circulation from tissues and blood cells, with their concentrations increasing during infections and inflammatory conditions. Few studies have evaluated serum TLR levels in sarcopenia. Toll-Like Receptor 4 and 9 (TLR4,9) are pattern recognition receptors, because this receptor family is activated by recognising biological molecules with common structures or motifs. In mammals, there are at least ten types of TLRs responsible for extracellular and intracellular activities [12]. These receptors can recognise common shared motifs (PAMPs) of exogenous pathogens as well as common motifs of damage markers (DAMPs) formed as a result of stress and inflammation [13]. TLR 9 specifically recognises CpG motifs. These motifs can be found in bacterial and viral DNA as well as in our own mitochondrial DNA. Especially in chronic inflammatory conditions, mitochondrial DNA is released into plasma and serum as a result of cell stress and tissue damage and leads to activation of inflammation by using TLR 9 pathway with increasing serum levels [13]. In a study conducted by Zhen Fan et al. in 2022, it was reported that serum cell free mitochondrial DNA level was significantly higher in the group with sarcopenia than in the group without sarcopenia in a population of 105 patients undergoing haemodialysis as renal replacement therapy due to chronic renal failure [14]. Sarcopenia is an age-related syndrome marked by the loss of muscle mass and function, with unclear mechanisms. Extra cellular matrix (ECM) remodeling and fibrosis in skeletal muscle are significant changes in sarcopenia. Increased TLR9 expression in aged mice's skeletal muscle correlates with muscle fibrosis. Using TLR9 knockout (KO) mice, researchers found that eliminating TLR9 reduced muscle fibrosis and improved muscle function in aged mice. Thus, targeting TLR9 could be a potential therapeutic approach for sarcopenia in aging [15]. TLR 4 is activated by LPS (lipopolysaccharide) in the bacterial wall, especially in gram-negative bacteria, as well as by a pro-inflammatory endogenous agent called resistin, whose serum level increases in chronic inflammatory conditions with widespread inflammation in the body. The TLR family are members of the innate immunity. They are activated in many conditions from infection to chronic inflammation and tumoural immunity. Serum levels increase in the above-mentioned conditions [16]. In the light of this information, we examined serum TLR 9 and TLR 4 levels in a condition with inflammation, tissue damage and atrophy such as sarcopenia. As a result of the study, we found that serum TLR 9 levels were significantly higher in the sarcopenia group compared to the other group. We found that serum TLR 4 levels were higher in the sarcopenia group, but we did not find this statistically significant. At this point, it can be said that serum TLR 9 level plays an important role in the pathophysiology of sarcopenia, although increasing the number of samples may contribute to statistical significance in terms of TLR 4 (Fig. 1).

In studies on lipid mediators in acute inflammatory response, omega-3 PUFA-derived mediators (Resolvin E1, D-series resolvins and E-series resolvins such as protectins) show anti-inflammatory effects by secretion from inflammatory exudate and resorption of inflammation [17]. Resolvin E1 targets neutrophils, dendritic cells, macrophages, platelets and adhesion molecules. In animal models, Resolvin E1 has been shown to have a strong protective effect against leukocyte-mediated tissue damage and excessive proinflammatory gene expression in the pathogenesis of inflammatory diseases. Recent studies have shown that Resolvin E1 plays a role in the etiopathogenesis of inappropriate inflammatory responses such as rheumatoid arthritis, multiple sclerosis, bronchial asthma, retinopathies, periodontal diseases and inflammatory bowel disease [18–20]. Luke A. Baker et al. observed that myoblasts in the cell culture underwent atrophy as a result of the inflammatory environment created by applying lipopolysaccharide (LPS) to the cell line in the experimental model they created with the C2C12 murine myoblast cell line in 2017, and that this effect was initially regressed when an omega 3 derivative called Reolvin E1 was applied to the cell culture [21]. In our literature review, although there are many publications on the measurement of serum levels of Resolvin E1 in various patient groups other than sarcopenia and that this level can be used in disease activity, pathogenesis and diagnosis, only a small number of studies on sarcopenia were found in experimental cell culture conditions, and these studies were carried out in order to observe the effects of reversing the effects developed in the inflammatory environment created as in the study mentioned above. In addition, different results have been found between serum levels and diseases. For example, in a study by Farhad Salari et al. serum levels of Resolvin E1 were found to be significantly higher in patients with allergic rhinitis than in healthy individuals [22]. In conclusion, the increase of Resolvin E1 was thought to be an indispensable effort of the immune system to actively resolve allergic airway inflammation. In a study conducted by Song et al. in 2021, it was found that serum Resolvin E1 levels in Hashimoto's patients were lower than in the healthy group [23]. In summary, when the studies in the literature are evaluated, it can be said that the serum level of Resolvin E1 may increase as a mediator trying to reverse the inflammation that occurs in inflammatory conditions or the serum level may decrease and contribute to the existing inflammation. In our study, we can interpret the higher level of RvE1 observed especially in the sarcopenia group as a kind of reverse response to increased inflammation.

The SARC-F questionnaire was developed by Malmstrom et al. in 2013 as a possible rapid diagnostic test for sarcopenia [24]. A score of 4 or higher on the SARC-F questionnaire was considered predictive of sarcopenia and its adverse consequences. After the publication of EWGSOP2, translation of SARC-F into world languages and validation studies were initiated. In the literature, validation studies suggesting the use of SARC-F as a screening test are predominant [25, 26]. In a study in which 207 people were included and the diagnosis of sarcopenia was evaluated according to EWGSOP, when the SARC-F ≥ 4 points was accepted as screening positive, the sensitivity for sarcopenia was found to be 25%, and specificity 81%. The low sensitivity of SARC-F has paved the way for alternative studies. There are studies showing that the sensitivity of SARC-F for sarcopenia can be increased by adding measurements such as BMI and calf circumference to the five questions [27, 28]. In addition, there are studies that reduced the number of questions in the SARC-F questionnaire from five to three. In a study by Woo et al. involving 4000 people over 65 years of age, it was suggested that SARC-F be reduced to three questions (strength, stair climbing and assistance in walking) [29]. As can be seen, there are studies on the translation and validity of SARC-F into different world languages, as well as studies aiming to modify the questionnaire and increase sensitivity. It revealed low sensitivity but high specificity with all sarcopenia definitions. Sensitivity and specificity were higher for muscle function tests reflecting its inquiry and input on functional measures. SARC-F is a good screening test to exclude muscle function impairment and sarcopenia for functional measures. In clinical practice, EWGSOP2 recommends the use of the SARC-F questionnaire to find persons at risk of sarcopenia.

The association between the risk of malnutrition and the incidence of sarcopenia is supported by the physiopathological link between these two conditions, in which certain features of malnutrition (low weight, unintentional weight loss, low macronutrient intake) potentiate the components leading to sarcopenia, such as loss of muscle strength and muscle mass, and reduce physical performance tested by various mechanisms [30]. In our study, although there was no malnutrition in both groups, we observed that the sarcopenia group had significantly lower BMI, MNA-SF and MNA-LF scores than the non-sarcopenia group. In addition, in order to see the effect of primary sarcopenia more clearly, we tried to keep our patient population away from comorbidities. As much as possible, people with less chronic diseases and drug use were included in the study. At this point, the Charlson Comorbidity Index, the number of chronic diseases and the number of medications used were similar in both groups and were observed at acceptable low levels.

In our study, when SARC-F and TLR 9 were compared, we found that both had similar sensitivity, specificity and AUC values for the diagnosis of sarcopenia. This result supports that the SARC-F questionnaire should be used to screen for sarcopenia as recommended by EWGSOP2. An important point to note is the difficulty of administering this questionnaire in patients with physical, mental and psychiatric disorders. In addition, since SARC-F is a self-report questionnaire, it is open to manipulation. In this case, the use of serum TLR 9 level, which is an objective value for sarcopenia, may be one of the options.

### Limitations

Case–control study design is the limitations of this study.

### Strengths

This is the first study to examine the relationship between primary sarcopenia and serum TLR4, TLR9 and Resolvin E1 levels and to compare the biomarkers with SARC-F.

## Conclusion

This study showed that primary sarcopenia was associated with serum TLR 9 level and SARC-F score. SARC-F and serum TLR 9 level showed similar diagnostic accuracy for sarcopenia. Since SARC-F is a self-report questionnaire, it is not as objective as serum TLR 9 level. Especially in cases where it is difficult to find cases with SARC-F (such as physical, mental or psychiatric disorders), assessment of serum TLR 9 level may be one of the options. This study supports the widespread use in daily practice of the SARC-F questionnaire recommended by the EWGSOP2 in 2019 for sarcopenia screening, as serum TLR 9 level cannot be used routinely and SARC-F can screen similarly to this biomarker. In addition, it would not be wrong to say that TLR 9 may play an important role in the pathogenesis of sarcopenia and functional studies in this field will provide diversity in terms of treatment options.

## Supplementary Information

Below is the link to the electronic supplementary material.

Supplementary file1 (DOCX 65 KB)

Supplementary file2 (DOCX 29 KB)

Supplementary file3 (DOCX 16 KB)

## Data Availability

Data will be made available on request.
