# Supplementary material for: Insights into geriatric health: primary sarcopenia and innate immunity dynamics, examining SARC-F, serum TLR 4, TLR 9, and resolvin levels
Source: Intern Emerg Med. 2024 Jun 23;19(7):1867–75. doi: 10.1007/s11739-024-03678-5 (PMC11467011; doi:10.1007/s11739-024-03678-5)
Supplement: Supplementary file 1 — Supplementary file1 (DOCX 65 KB) [file 11739_2024_3678_MOESM1_ESM.docx]

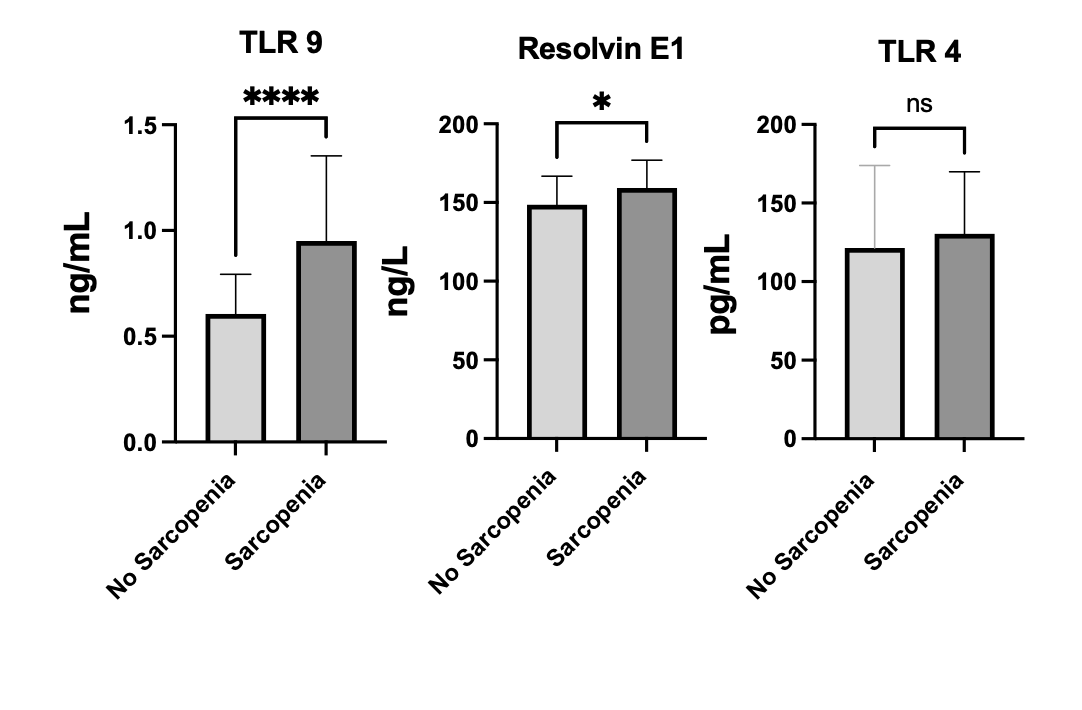


**Supplementary Fig 1. Comparison of serum TLR 4, TLR 9 and Resolvin E1 levels in sarcopenia and non-sarcopenia groups.**

**** p<0.001 * p=0.040 ns ( non significant) p=0.742

Data are shown as median (25th percentile-75th percentile)

TLR (Toll Like Receptor)
