# Supplementary material for: Insights into geriatric health: primary sarcopenia and innate immunity dynamics, examining SARC-F, serum TLR 4, TLR 9, and resolvin levels
Source: Intern Emerg Med. 2024 Jun 23;19(7):1867–75. doi: 10.1007/s11739-024-03678-5 (PMC11467011; doi:10.1007/s11739-024-03678-5)
Supplement: Supplementary file 2 — Supplementary file2 (DOCX 29 KB) [file 11739_2024_3678_MOESM2_ESM.docx]

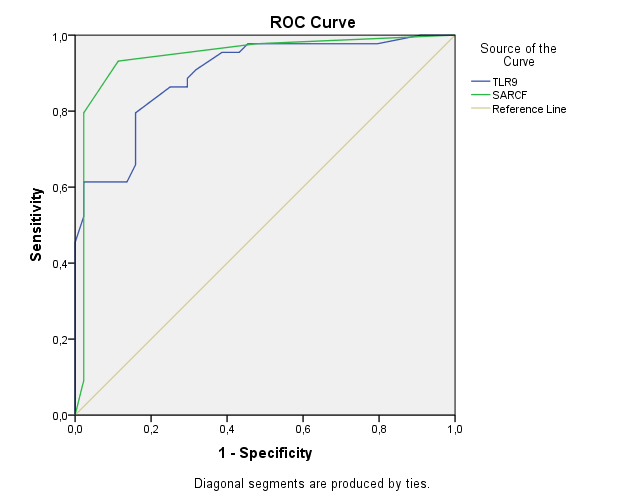


**Supplementary Fig 2.** Receiver operating characteristic (ROC) curve analysis for TLR 9 and SARC-F for accuracy of sarcopenia. Area under the ROC curve (AUC) for TLR9 0.896 (*p* <0.001), SARC-F 0.943 (*p* <0.001).
