# Supplementary material for: Insights into geriatric health: primary sarcopenia and innate immunity dynamics, examining SARC-F, serum TLR 4, TLR 9, and resolvin levels
Source: Intern Emerg Med. 2024 Jun 23;19(7):1867–75. doi: 10.1007/s11739-024-03678-5 (PMC11467011; doi:10.1007/s11739-024-03678-5)
Supplement: Supplementary file 3 — Supplementary file3 (DOCX 16 KB) [file 11739_2024_3678_MOESM3_ESM.docx]

**Supplementary Table 1. Comparison of some serum biochemical values in the group with and without sarcopenia.**

|  | **Sarcopenia** | **No Sarcopenia** | **p** |
| --- | --- | --- | --- |
| Neutrophil Count* (10^3/μL) | 2.39±1.30 | 1.95±1.00 | 0.084 |
| Monocyte Count* (μL) | 581.39±217.40 | 545.45±164.88 | 0.387 |
| Platelet Count* (μL) | 256604.65±68735.21 | 250022.72±61574.86 | 0.639 |
| Lymphocyte Count* (μL) | 1976.74±963.58 | 2122.72±712.95 | 0.423 |
| Haemoglobin* (g/dL) | 12.59±1.25 | 13.30±1.22 | **0.010** |
| Creatine* (mg/dL) | 0.81±0.19 | 0.87±0.27 | 0.230 |
| Vit-D* (pg/L) | 25.81±11.97 | 25.07±12.10 | 0.777 |
| B12* (ng/L) | 454.92±330.77 | 420.72±199.99 | 0.560 |
| Folic Acid* (ng/mL) | 7.96±3.43 | 8.33±4.14 | 0.657 |
| CRP* (mg/L) | 3.92±4.92 | 4.64±6.74 | 0.574 |
| Sedimentation Rate* (mm) | 15.93±9.29 | 17.00±10.45 | 0.616 |
| Fasting Glucose* (mg/dL) | 96.55±19.46 | 99.84±23.80 | 0.484 |
| HbA-1c* (%) | 6.03±1.33 | 5.96±0.72 | 0.741 |
| Ferritin* (ug/L) | 74.20±49.21 | 80.28±56.74 | 0.595 |
| Transferrin Saturation* (%) | 20.56±8.11 | 23.73±9.33 | 0.095 |

Statistically significant p values are indicated as bold

*mean±standard deviation

CRP (C- Reactive Protein)
